# Meteorological Variables and Suicidal Behavior: Air Pollution and Apparent Temperature Are Associated With High-Lethality Suicide Attempts and Male Gender

**DOI:** 10.3389/fpsyt.2021.653390

**Published:** 2021-03-05

**Authors:** Andrea Aguglia, Gabriele Giacomini, Elisa Montagna, Andrea Amerio, Andrea Escelsior, Marco Capello, Laura Cutroneo, Gabriele Ferretti, Davide Scafidi, Alessandra Costanza, Gianluca Serafini, Mario Amore

**Affiliations:** ^1^Section of Psychiatry, Department of Neuroscience, Rehabilitation, Ophthalmology, Genetics, Maternal, and Child Health, University of Genoa, Genoa, Italy; ^2^Istituto di Ricovero e Cura a Carattere Scientifico Ospedale Policlinico San Martino, Genoa, Italy; ^3^Department of Psychiatry, Tufts University, Boston, MA, United States; ^4^Department of Earth, Environment, and Life Sciences, University of Genoa, Genoa, Italy; ^5^Department of Psychiatry, Faculty of Medicine, University of Geneva (UNIGE), Geneva, Switzerland; ^6^Department of Psychiatry, Azienda Ospedaliera Nazionale Santi Antonio e Biagio e Cesare Arrigo Hospital, Alessandria, Italy

**Keywords:** suicide attempt, environmental parameters, apparent temperature, air pollution, hospitalization, suicide, suicidal behavior

## Abstract

This study analyzed the impact of meteorological variables and high-lethality suicide attempts (HLSA) to assess a potential time shift of HLSA affected by climate evolution to predict the suicide attempt cases over different periods of the year. After attempting suicide, 225 subjects were admitted to the emergency ward of the IRCCS Ospedale Policlinico San Martino and later to the psychiatric unit from March 2016 to July 2018. Socio-demographic and clinical characteristics as well as the meteorological variables were collected. The Mann-Kendall test as well as redundancy and cross-correlation analyses were performed to analyze the trends, statistically correlations, and correspondence of the trends, respectively between suicidal behaviors and climatic factors. Sixty-seven (29.8%) committed a HLSA. Our findings indicate a significant association between HLSA and male gender and apparent temperature with a strong correlation of 75% with a phase shift of −1 month. Solar radiation and air pollution (PM_2.5_) have a positive correlation of 65 and 32%, respectively, with a zero-time lag. Limitations include that the data are limited to a single hospital; psychological factors, or other clinical variables that could be ruled out as a trigger have not been considered. Meteorological variables may not mirror the temperature that the patient is exposed to due to the air conditioning systems. Exploring those environmental factors associated with HLSA in a more detailed manner could lead to early intervention and prevention strategies for such distressing admissions.

## Introduction

Suicide is a preventable public health issue causing more than 800.000 annual deaths globally; it is the second leading cause of death among 15- to 29-years-olds worldwide (1).

Climate changes based on extreme temperatures, floods, drought, tornadoes, hurricanes, and wildfire can lead distress symptoms, suicidal behaviors, and psychiatric disorders in the general population. Global warming fits into this context with possible negative influence of environmental parameters on mental health and psychiatric disorders through both indirect and direct factors (2, 3). Changes in temperature, especially during the transition between winter and spring, can have remarkable consequences on suicidal behavior trends. Mental disorders and rates of attempted and complete suicidal behaviors have been studied for years to understand their trends and triggers and identify the influence of external risk factors such as social, economic, and environmental factors (4–6). In a multicenter study, a strong association was reported between climate effects vs. economic factors on suicidal behavior. The authors found that suicidality in Europe followed the climate/temperature change, which was from south to north-east (6). On the contrary, it has been hypothesized that higher lithium levels in drinking water may be associated with a reduced risk of suicidal behaviors in the general population (7–9).

Therefore, some research suggested that environmental conditions might be potentially important trigger factors. Specifically regarding meteorological variables, a positive correlation between seasonality (10–14) or sunlight exposure, also called photoperiod (15, 16), or latitude (17), and suicidal behaviors has been shown. Recently, a significant association between hours of sunshine and violent completed suicides (18, 19) or high-lethality suicide attempts (HLSA) (20, 21) was reported. These findings were not confirmed by Veisani et al. (22) who showed seasonal trends in spring and autumn of suicides by violent methods and spring and summer for what concerns non-violent suicides.

The mechanisms underlying this association are still unclear and need further investigation. Therefore, other environmental variables such as increased ambient temperature or air pollution could be implicated in this complex phenomenon. A potential role of ambient or apparent temperature on suicidal behaviors has been studied with controversial results (15, 16, 23–28). Several data, however, showed that a 26.6–60% of variation in suicidal behaviors could be explained by temperature fluctuations (29–31) but not with sunlight exposure, especially in completed suicide (16). Some researchers found a relationship between the warmest temperatures and suicide rates, mostly involving older subjects (32) and men relative to females (33) while others (34) found no association between weather variables and suicides in Colombia highlighting the absence of association in a tropical country where seasonal changes are not marked.

Among the environmental factors, air pollution and ozone could also have a detrimental impact on mental health. For example, Bernardini et al. (35) considered daily admissions to psychiatric emergency services in two Italian hospitals over a period of 2 years and highlighted that exposures to ozone may be associated with increased psychiatric admissions. Recently, several studies reported that higher levels of particulate matters (PM_2.5_ and PM_10_) were linked to an increase in suicidal behaviors (attempts and deaths) worldwide (36–44), demonstrating both short- and long-term effects. Nevertheless, other studies report the absence of this association (34, 45). Although the specific mechanisms linking air pollution and suicide are uncertain, a growing body of research does suggest that the link is plausible.

Genoa has a population of about 600.000 inhabitants with an area of 240 km^2^. Its territory is located in the Liguria Region, and it has typically a Mediterranean climate with relatively temperate winters (mean temperature 8°C in January) and hot summers (mean temperature 24°C in July). Rains are concentrated in autumn (annual mean precipitation 1,100–1,300 mm) (46). Genoa territory is ringed to the north by rugged mountains with wide gaps between them, which channel and control the movement of air masses. Therefore, despite the mild climate, these morphological characteristics combined with the fact that Genoa is located at the apex of the Ligurian sea on the northern side of one of the most active cyclogenetic areas in Europe, lead to frequent periods of unstable weather (47) that could affect the mental health of the population.

In recent decades, several climate changes have been reported in Genoa. For example, Faccini et al. (46) analyzed Genoa weather data between 1833 and 2014 and found that the annual mean temperature and rainfall rate (the ratio of annual rainfall and the number of rainy days) had a statistically positive trend (+0.15°C yr^−1^ and +0.28 mm yr^−1^, respectively) while rainy days had a negative trend (−0.43 d yr^−1^) suggesting a shift toward more extreme weather.

Data from the Italian National Institute of Statistics (ISTAT; www.istat.it) suggested that the province of Genoa has attempted and completed suicide rates of 17.2 and 10.0 cases per 100.000 inhabitants, respectively. On the contrary, the city of Genoa had a mean of 23 cases per year of death by suicide from 2007–2017; the victims were mostly males (71%).

To the best of our knowledge, no Italian study has yet reported data about meteorological parameters (including temperature and air pollution) and HLSA. Therefore, the aim of this study was to analyze the impact of several meteorological parameters such as air temperature, rain, solar radiation, and air pollutants, recorded by different measurement stations in Genoa (north-west Italy), on suicidal behaviors (especially HLSA). Second, we assessed a potential time shift of HLSA in a Mediterranean city characterized by a mild climate but affected by climate evolution such as the increase of flood events and temperature to predict the suicide attempt cases during the different periods of the year.

## Materials and Methods

### Sample

We recruited individuals consecutively admitted for a suicide attempt (225 cases) and referred to the Emergency Department of IRCCS Ospedale Policlinico San Martino (Genoa) over a period of 2 years (from March 2016 to July 2018). This hospital works as for the whole Liguria region and the northwestern Italian area. It is the main hospital in Genoa and it is located in the eastern part of the city with a catchment area that extends to the neighboring municipalities. After clinical and medical evaluation, these individuals were admitted to the Psychiatric Unit.

The inclusion criteria were: (a) being hospitalized in an emergency psychiatric ward for a suicide attempt; (b) age > 18 years; and (c) the willingness to participate in the study by signing a written informed consent. The exclusion criteria were: (I) pregnancy or having just given birth; (II) having a positive history of acute neurological injuries such as neurodegenerative illnesses, intellectual disability, and loss of consciousness related to the presence of severe neurological conditions; (III) the assumption of melatonergic or lipid-lowering agents; (IV) the presence of acute or severe medical condition; and (V) the refusal or inability to provide a valid consent prior to participation in the study.

All participants received a detailed explanation of the study design and written informed consent was obtained from all respondents according to the guidelines provided in the current version of the Declaration of Helsinki. The study design was approved by the local Ethical Review Board.

### Assessment and Procedures

Socio-demographic and clinical characteristics were investigated through the standardized clinical chart and lifetime computerized medical record used in our Psychiatric Unit. Age, gender, marital status, occupation, education level, suicide method, and psychiatric diagnosis according to the Diagnostic and Statistical Manual of Mental Disorders, fifth edition (DSM-5) criteria (48) at discharge were recorded and grouped as in previous research (49, 50). All available information have been cross-referred and revised by two senior psychiatrists (MA and GS).

Silverman's operative definition of “suicide attempt” was used in the present study: a kind of suicide-related behavior classified as a suicidal act and characterized by self-inflicted, potentially injurious behavior with the non-fatal outcome for which there is evidence – either explicit or implicit – of the intent to die (51). Moreover, our definition involved the presence of a lethal intent that may be of varying intensity but needs to be present in the decision to carry out the suicidal act (52).

The cases of suicide attempts were divided into two subgroups based on the severity of the suicidal behaviors. The term “suicidal lethality” has not yet been defined comprehensively and, in our study, we adopted the concept of lethality as defined by Shneidman's (53) and Joiner's (54) criteria even used in recently published reports (20, 21, 55, 56). Methods of suicide attempt were dichotomized in terms of lethality. Therefore, a HLSA was defined as a suicide attempt that warranted hospitalization for at least 24 h and either treatment in a specialized unit (including intensive care unit, hyperbaric unit, or burn unit), surgery under general anesthesia, or extensive medical treatment (beyond gastric lavage, activated charcoal, or routine neurological observations), including antidotes for drug overdoses, telemetry, or repeated tests or investigations. Conversely, a low-lethality suicide attempt (LLSA) was defined as a suicide attempt that did not meet these criteria (20, 21, 55, 56).

The environmental parameters included air mean temperature (T, °C), precipitation (P, mm), wind speed (WS, m sec^−1^), and relative humidity (RH, %) data recorded by the Meteorological Observatory of the University of Genoa (MO-UNI). Solar radiation (SR, J cm^−2^) and atmospheric pressure (AP, hPa) data were recorded by the weather station of the Regional Agency for the Protection of the Ligurian Environment (W-ARPAL). Particulate matter with diameter ≤ 10 μm (PM_10_) and with diameter ≤ 2.5 μm (PM_2.5_; μg m^−3^) as well as CO (mg m^−3^), NO_2_ (μg m^−3^), and benzene (μg m^−3^) were measured *via* an air pollution detection station of ARPAL (P-ARPAL) (Genoa – Latitude: 44°24′40″ N; Longitude: 008°55′58″ E).

### Statistical Analysis

Starting from data recorded by the weather station, the apparent temperature (AT) was calculated according to the following formula:

(1) $$\begin{array}{l} AT=T+0.33\;*\;Wp-0.70\;*\;WS-4.00 \end{array}$$

where *T* is the air temperature, *WS* is the wind speed, and *Wp* is the water vapor pressure, calculated as following:

(2) $$\begin{array}{l} Wp=RH/100\;*\;6.105\;*\;exp\left(17.27\;*\;T/\left(237.7+T\right)\right) \end{array}$$

where *RH* is the relative humidity.

Distributions of monthly cases of attempted suicide and environmental parameters were statistically tested to find eventual significant trend: the Mann-Kendall trend test with a *p*-value = 0.05, was applied by the statistical software ProUCL (v. 5.1; U.S. Environmental Protection Agency).

Redundancy analysis (RDA) (57) was applied to data on suicide attempts (HLSA, LLSA, and total cases). Environmental parameters (T, P, WS, RH, SR, AP, PM_10_, PM_2.5_, CO, NO_2_, benzene) were used to find potential statistically correlations. RDA investigates the presence of relationships between the explanatory variables (meteorological parameters) and the response variables (the lethality of suicide attempts and gender) of a data matrix. Given the diversity between the ranges of the considered parameters, the data were standardized before the analysis. RDA was performed using the Brodgar software (Highland Statistics Ltd., v. 2.7.5, 2017).

The cross-correlation analysis was applied to the number of cases (total cases, HLSA, and LLSA) and to the environmental parameters that in the RDA showed a correlation with suicide attempts. The cross-correlation is a mathematical function that allows the comparison of two series by measuring the level of similarity and the time lag between them. To facilitate the interpretation of the cross-correlation function, the systems are normalized and ranges between −1 and +1. If the value of the normalized cross-correlation is equal to 1 it means that the two series are identical (100% positive correlation). If the series are not similar, then it tends to approach 0. If a negative correlation is found, then they are anticorrelated and the value is −1 (100% negative correlation). The time to which the maximum value of the cross-correlation function represents the time lag between the two series. Cross-correlation was performed using PITSA software package (58).

## Results

### Characterization of the Total Sample

We included 225 subjects admitted for suicide attempt, of which 29.8% (*N* = 67) presented a HLSA and 70.2% (*N* = 158) a LLSA. One hundred seventy five (75.6%) were females, half of the individuals was unmarried, and about a third was actively working. Distributions of the different diagnoses did not show statistically significant trends, except for “other diagnoses”, which decreased trend. The greatest number of cases are concentrated in the spring-summer months for bipolar disorder. The most commonly used method of suicide attempts was drug intoxication. Further details about sociodemographic and clinical characteristics are summarized in Table 1.

**Table 1:** Sociodemographic and clinical characteristics of the total sample (*N* = 225).

**Gender (female), *N* (%)**	**170 (75.6)**
Age, *N* (%)	
<40 years	82 (36.4)
40–60 years	72 (32.0)
>60 years	71 (31.6)
Education level, *N* (%):	
Primary school (1–5 years of school)	9 (4.0)
Secondary school (6–8 years of school)	66 (29.3)
High school (9–13 years of school)	112 (49.8)
Graduation and post (>14 years of school)	38 (16.9)
Marital status, *N* (%)	
Unmarried	109 (48.4)
Married	43 (19.1)
Separated/divorced	47 (20.9)
Widowed	26 (11.6)
Working status, *N* (%)	
Unemployed	150 (66.7)
Employed	75 (33.3)
Primary diagnosis, *N* (%):	
Bipolar and related disorders	72 (32.0)
Schizophrenia and related disorders	21 (9.3)
Depressive disorders	72 (32.0)
Others	60 (26.7)
Lethality of suicide attempt, *N* (%)	
High-lethality suicide attempt	67 (29.8)
Low-lethality suicide-attempt	158 (70.2)
Modality of suicide attempt, *N* (%)	
Drug intoxication	147 (65.3)
Cuts	35 (15.6)
Defenestration	17 (7.6)
Weapon	1 (0.4)
Stabbing	3 (1.3)
Burn/gas/caustic	14 (6.2)
Strangling	8 (3.6)

### Characteristics of Environmental Parameters

All environmental parameters showed typical values and variations in the Mediterranean climate that characterizes the Genoa area. However, the beginning of 2018 was characterized by some peculiarities from the meteorological point of view: a relatively warmer January (mean temperature of 11.2°C in January 2018 compared to 7.5°C in January 2017), the shift of the minimum winter temperature in February, and a spring with high cumulative precipitation, especially in March 2018 (178.8 mm).

**Figure 1:**
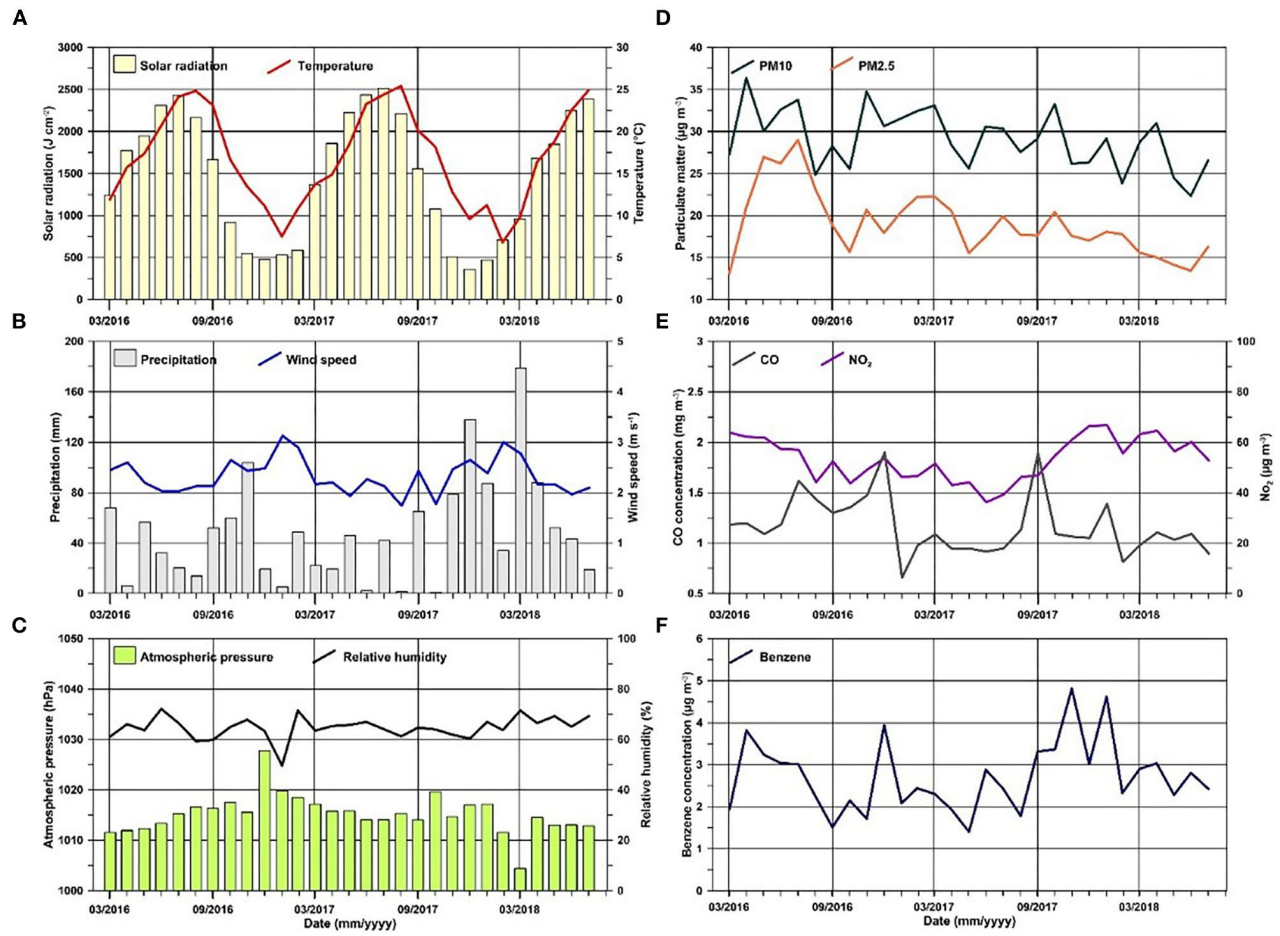
Monthly distribution of **(A)** solar radiation (pale yellow bars) and temperature (red line); **(B)** precipitation (gray bars) and wind speed (blue line); **(C)** atmospheric pressure (green bars) and relative humidity (black line); **(D)** particulate matter concentrations (PM_10_-dark green line and PM_2.5_-orange line); **(E)** CO (gray line) and NO_2_ (violet line) concentrations; **(F)** benzene concentrations (dark blue line).

Distributions of all the environmental parameters analyzed are reported in Figure 1. Only temperature and solar radiation show a clear seasonality in the period typical of the mean latitude.

Among the environmental parameters, only CO, PM_10_, and PM_2.5_ have statistically significant trends and all three have decreasing trends.

### Influence of Environmental Parameters

Several tests with RDA, combining suicide lethality and gender (response variables) with the environmental parameters (explanatory variables), were made.

**Figure 2:**
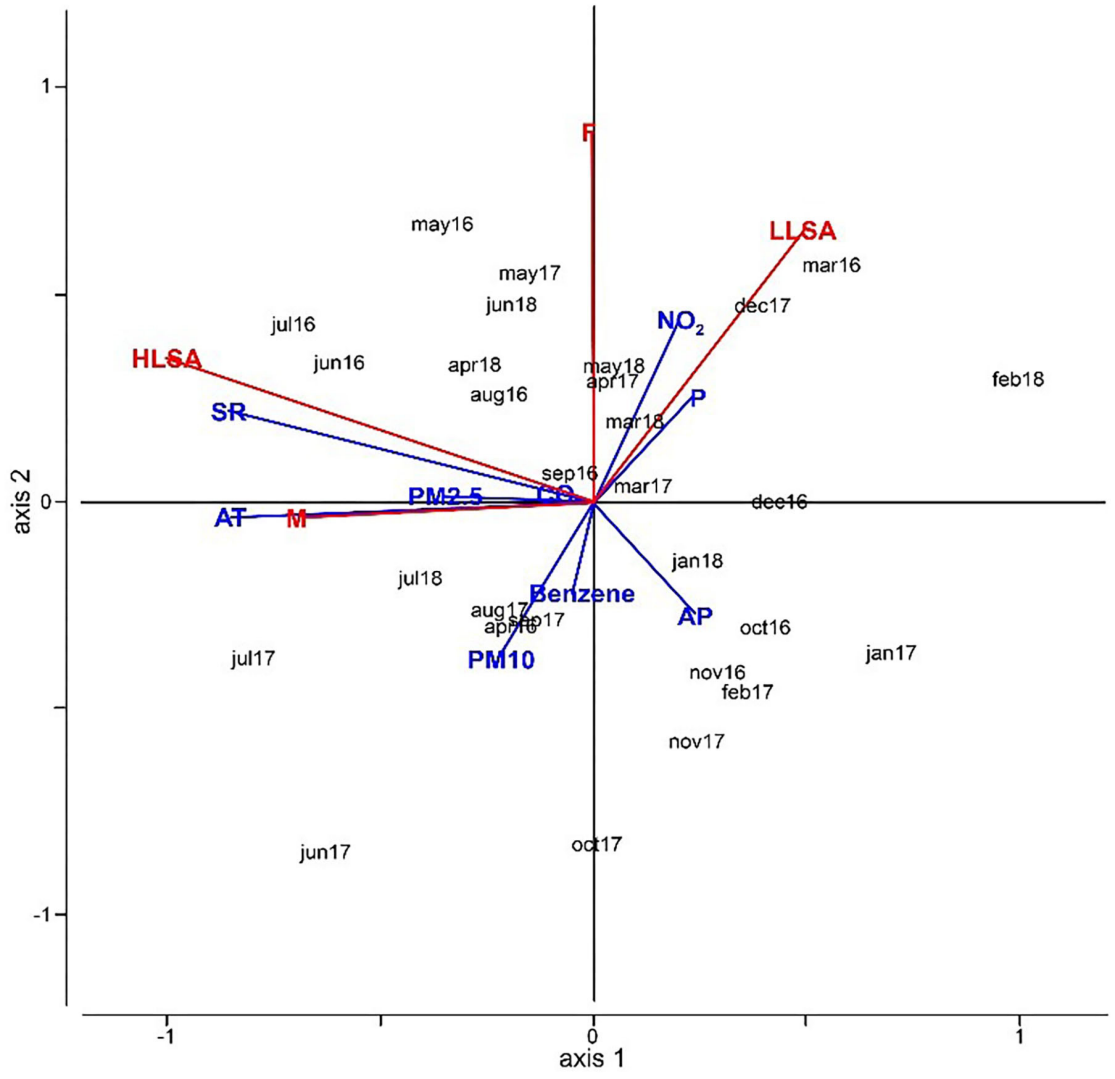
RDA analysis combining suicide lethality and gender (response variables) with environmental parameters. The sum of all canonical eigenvalues is 0.42.

The Figure 2 shows the results of RDA carried out with HLSA, LLSA, gender, and environmental parameters. These results are obtained by using AT instead of the individual parameters WS, T, HP, and WP to better summarize the results. Furthermore, AT has been proven to characterize the physiological experience better than just the temperature or the other parameters alone [Niu et al. (27)]. In our case, SR, AT, and PM_2.5_ are very closely related to the HLSA and male subjects while LLSA and females are correlated to NO_2_ concentration and precipitation. Therefore, most findings demonstrate how SR and AT can influence the distribution of cases.

Following the RDA results, we applied cross-correlation analysis between pairs of parameters to verify the degree of relationship between an environmental parameter and one relating to suicide attempts and highlight the time lag between the two; hence, there is a delay in response in patients following the environmental stimulus.

**Figure 3:**
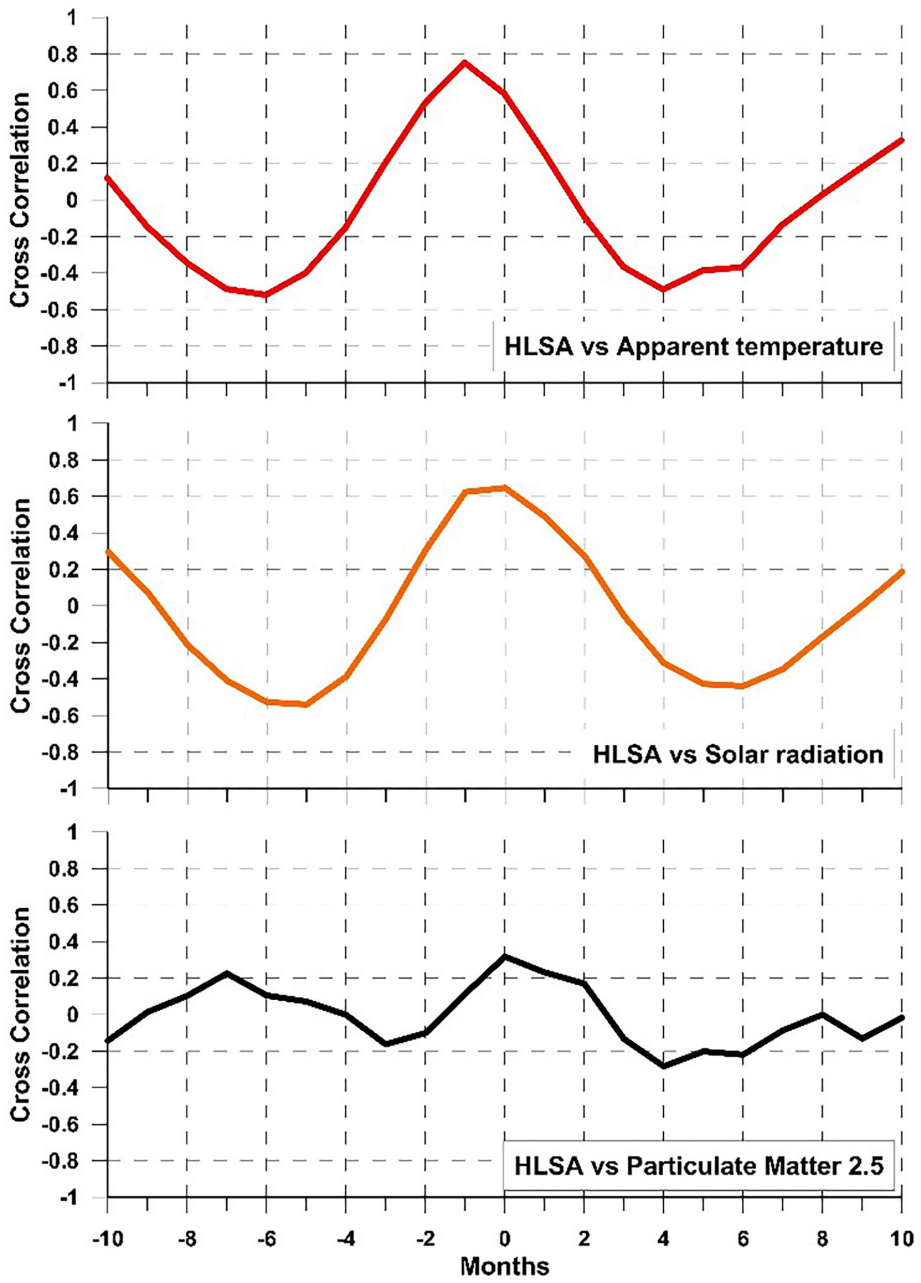
Cross-correlation results: HLSA *vs*. apparent temperature (red); HLSA vs. solar radiation (orange); HLSA vs. PM_2.5_ (black).

Considering HLSA and AT (Figure 3), a positive and strong correlation of 75% was found with a phase shift of −1 month, i.e., suicide attempts peak with HLSA 1 month before reaching the AT maximum in the phase of AT increase. Considering HLSA and solar radiation, a positive correlation of 65% is obtained with a zero-time lag: therefore, the monthly increase in solar radiation corresponds to a tight monthly increase in cases of HLSA and a decrease in light corresponds to a decrease in violent suicide attempts. Between HLSA and PM_2.5_, there is a 32% of correlation without time shift. Therefore, the increase in PM_2.5_ corresponds with an increase in suicide attempts albeit with a relatively low degree of correlation.

## Discussion

To our knowledge, this is the first Italian study investigating the correlation between HLSA and several meteorological variables. We found a significant association between three weather parameters (apparent temperature, solar radiation and PM_2.5_) and both HLSA and male gender. This is confirmed by Dumenčić et al. (33) that demonstrated, in a sample of 478 suicide attempts in Croatia over 10 years, a significant influence of temperature on males more than females.

It has been widely reported in literature that these variables influence not only the mental health but also suicidal behaviors. Nowadays, with global warming and a greater focus on climate issues, several efforts have been made in this direction. A recent meta-analysis (16) demonstrated a significant positive association between temperature rises and incidence of suicidal behaviors, while the potential role of solar radiation is more controversial (59), confirmed previously (15, 16, 23–28, 60, 61). The innovative aspect of our study was the significant association of meteorological variables with HLSA.

As observed by Postolache et al. (62), several neurotransmitters (e.g., dopamine, norepinephrine, and serotonin) affect both mood and thermoregulation. Specifically, serotonin is a neurotransmitter regulating emotion that is sensitive to weather variability and light exposure, so springtime changes in expression might be associated with increased suicidal behavior (63, 64). Regarding biochemical mechanisms underlying this phenomenon, it has been proposed that warming temperatures enhance the responsiveness of 5-HT_2A_ receptors of the central nervous system (CNS) (65). The activation of 5-HT_2A_ receptors increases the sympathetic outflow to brown adipose tissue (BAT) (66) and hyperactivity of BAT impairs heat tolerance, and it could enhance the risk of suicidal behaviors. As proposed by Helama et al. (29): a potential association exists between psychiatric condition (i.e., anxiety and/or depressive symptomatology or disorders leading to suicidal behaviors) and altered transmission in the CNS projecting from BAT through the hypothalamus to the periaqueductal gray areas. Regarding structural brain findings, some authors have recently reported that individuals who commit HLSA could show larger pre-frontal cortex, caudate, and insula volumes compared to LLSA and individuals without suicidal behaviors. Furthermore, suicide attempters with familiarity for suicidal behavior reported smaller volumes in temporal regions, dorso-lateral pre-frontal cortex, and putamen. These cerebral structures play a role in cognitive process like decision making, inhibition, emotional dysregulation, and risk perception, and when compromised because of several factors (i.e., heat, climate change, sleep-deficiency), they could lead to more impulsive decisions and acts (67). Lastly, based on the assumption that similar areas of the insular cortex are activated during recall of feelings and sensation of pain and temperature (68), we assumed a link between the perception of significant heat and emotional correlation of discomfort, which could lead to suicidal behaviors in subjects at risk. Maes et al. (69) found a trend in L-tryptophan levels inversely related to the seasonal variation of violent suicides in the Belgian population. Furthermore, a correlation between peripheral dysfunctions of serotoninergic transmission, assessing metabolic, such as cholesterol and triglycerides, and hematologic, such as platelet-lymphocyte ratio and mean platelet volume, parameters and HLSA was demonstrated by recent findings (55, 56). A very recent review on the complex phenomenon of suicidal behaviors, conducted by Mann et al. (67) confirmed these findings and concluded that there is deficient serotonin release in HLSA. This is not likely due to serotonin biosynthesis, but seems to be associated with up-regulated serotonin_1A_ (5-HT_1A_) auto-receptors located on serotonin neuron cell bodies and proximal dendrites (70) or postsynaptic cortical serotonin_2A_ receptors (5-HT_2A_) that correlate with lifetime aggression severity (71). Serotonin dysfunction is more prominent in high-lethality suicidal behavior.

In our study, we found a positive correlation between HLSA and PM_2.5_. Recently, several studies confirmed our findings, reporting short- and long-term effects of higher levels of PM_2.5_ and PM_10_ on suicidal behaviors, including attempted and completed suicides (36–44). However, the concentration and composition of air pollutants and population-level features (i.e., age and race) vary across countries.

The possible biological explanation of the observed association between air pollution and HLSA has not been clarified. A link between suicidal behaviors and inflammation was recently reported (6). Therefore, it is hypothesized that PM_2.5_ and PM_10_ could induce systematic inflammation/oxidative stress or impair hippocampal neurogenesis and neurotrophic factor expression, leading pollutants to play a causative role in the occurrence of psychological problems or suicide attempts. So, PM_2.5_ enters the olfactory bulb and reaches the basis nuclei (substantia nigra and striatum), causing a release of pro-inflammatory factors and cytokines, such as tumor necrosis factor-alpha (TNF-α), interleukin-6 (IL-6) and interleukin-1beta (IL-1β). Besides, air pollution causes a chronic activation of brain capillaries, astroglia, and, particularly, microglia, leading to inflammation and oxidative stress (72). Recently, Baharikhoob and Kolla (73) proposed the following relationship between microglial dysregulation and suicidal behaviors: pro-inflammatory cytokines (i.e., IL-1β, TNF-α, interferon-gamma (IFN-γ), and IL-6) would provoke neurotoxicity by increased glutamate (dependent on loss of inhibition in glutamatergic neurons). This hypothesis is confirmed by literature evidence, in which a significant correlation between higher IL-6 levels and suicide attempters' impulsivity was demonstrated (74). Furthermore, a recent study reported that high IL-6, IL-10, and C-reactive protein levels and activated microglia-related alterations were associated with greater suicidal ideation severity (75). Therefore, neuroinflammation seems to be one of the most involved factors in the pathogenesis of suicidal behavior and neurotoxicity and disrupted blood-brain barrier leads to impulsive, aggressive, and self-destructive behaviors.

Our study has several limitations. First, our data are limited to a single hospital. Second, psychological factors (e.g., life events, no compliance to treatment, and poor social support) that could be ruled out as triggers have not been considered. Furthermore, the effect of other clinical variables has not been investigated. Therefore, the generalization from our results should be made with caution. No structured interviews were performed to formulate psychiatric diagnoses and evaluate the lethality of suicide attempts (such as the Columbia Suicide Severity Rating scale). We preferred to rely upon Shneidman's and Joiner's definitions of lethality, although we acknowledge that using a specific instrument might provide adjunctive relevant data. Lastly, meteorological variables measured outdoor might not mirror the temperature the patient is exposed to, due to the increasing number of air conditioning systems. This might be responsible for the well-known ecological regression bias.

In conclusion, we found that apparent temperature, solar radiation and PM_2.5_ exposures were associated with an increased risk of HLSA in the Italian population. Our study extended prior results of an acute effect of seasonality and photoperiod on HLSA. Based on our findings, an increase in HLSA is expected within a month after a heatwave. Therefore, clinicians should monitor strictly high-risk subjects when hot temperatures occur and between the seasons (i.e., from spring to summer), playing a key role in predicting and preventing risky behaviors based on the knowledge of climatic variables. Furthermore, the correlation between multiple suicide attempters and climatic variables throughout the year is a crucial issue in the field of suicidology, assuming that a history of multiple attempters could be more vulnerable to heat stress. It is also necessary to evaluate the change in concentrations of air pollutants, as well as the degree of health damage, especially in areas of the world where air pollution is highest, and search potential biochemical mediators that could explain the greater influence of climatic and environmental factors in males than females.

Future prospective or cohort studies with a larger sample, exploring in a detailed manner these environmental factors, are warranted to further test the topic and clarify the impact of climatic factors on HLSA to reduce or prevent health damage, implementing early intervention for mental health promotion, especially focusing on vulnerable at-risk populations. Therefore, for suicidologists and their organizations, the potential rise in suicides is troubling and should lead to our support for those arguing for climate change policies. The organizations involved in suicide prevention should make public position statements, lending support to the growing demand for action on climate change (76).

## Data Availability Statement

The raw data supporting the conclusions of this article will be made available by the authors, without undue reservation, upon request.

## Ethics Statement

The study was reviewed and approved by the Ethics Committee (IRCCS Ospedale Policlinico San Martino). The patients/participants provided their written informed consent to participate in this study.

## Author Contributions

AAg and LC designed the study and wrote the first draft of paper. AE and AAm participated to the first revision of original article and revised the English language. GG, AC, and EM collected the data and managed the literature searches. DS, LC, and GF undertook the statistical analyses and wrote the result section. GS, MA, and MC supervised the study design, search strategy reviewed, and edited the original draft. All Authors contributed to and approved the final version of the manuscript.

## Conflict of Interest

The authors declare that the research was conducted in the absence of any commercial or financial relationships that could be construed as a potential conflict of interest.
